# Effect of Epoxy Resin on the Actuating Performance of Piezoelectric Fiber Composites

**DOI:** 10.3390/s19081809

**Published:** 2019-04-16

**Authors:** Xiaoyu Wang, Xi Yuan, Mingliang Wu, Feng Gao, Xuemei Yan, Kechao Zhou, Dou Zhang

**Affiliations:** 1 Beijing Spacecraft System Engineering, Beijing 100094, China; antlb@163.com (X.W.); gaofeng_cast@163.com (F.G.); yanxuemeiqq@sina.com (X.Y.); 2 State Key Laboratory of Powder Metallurgy, Central South University, Changsha 410083, China; wumingliang55@126.com (M.W.); zhoukechao@csu.edu.cn (K.Z.); 3 College of Chemistry and Chemical Engineering, Central South University, Changsha 410083, China

**Keywords:** Epoxy resin, viscosity, piezoelectric fiber composites, actuating performance

## Abstract

Piezoelectric fiber composites (PFC) have shown excellent performance in the areas of vibration control and deformation control. The viscosity and rigidity of the epoxy resin before and after curing, respectively, were very important factors that affected the performance of PFC. In this paper, Aradite 2020, DP 460, and DP 490 epoxy resins, with the viscosities of 0.15, 25.0, and 250.0 Pa·s, respectively, were employed to encapsulate the piezoelectric fiber composite. The PFC that was packaged with Araldite 2020 had the best free strain of 1420 ppm and tip displacement of 17.8 mm. DP 490 caused the lowest performance of PFC, due to the highest viscosity. When the environmental temperature increased from −40 to +80 °C, the free strain of PFC with Aradite 2020 increased at first and then decreased, reaching a maximum value of 1440 ppm at 30 °C, which was mainly related to the mismatch of the resin/ceramic thermal expansion coefficient.

## 1. Introduction

As one of the important smart materials, piezoelectric fiber composites (PFC) have shown excellent performance in the areas of vibration control, deformation control, structural health monitoring and energy harvesting [1,2,3,4]. PFCs exhibit good flexibility and large deformation capability after the piezoelectric fibers, and polyimide films with interdigital electrodes are laminated and composited by epoxy resin. Piezoceramics are easy to break and they have poor fatigue resistance due to the brittleness. Epoxy resin therefore plays an important role in the PFC. In general, the resin should fill in the gaps between the fibers, and after curing, the resin is able to transmit the stress and prevent the propagation of cracks when the crack is about to generate. The rigidity of epoxy resin could affect the performance of the composites. Viscosity is also an important parameter for epoxy and it prominently affected the adhesive property of the composites [5,6,7,8,9]. Pomázi et al. [10] mentioned that the viscosity of the polymer matrix was a key property during the production of composites by injection technologies. Hwang et al. [11] found that viscosity was an important cure-dependent property in controlling the curing reaction of the resin in the cure cycle design of epoxy resin.

Meanwhile, a very thin layer of resin functioning as the bonding layer between piezoelectric fiber and the electrode could weaken the effective electric field intensity that is applied on the piezoelectric fiber [12]. Wieland et al. [13] and Wu et al. [14] demonstrated that increasing the dielectric constant of the epoxy resin would significantly increase the driving ability of the piezoelectric fiber composite. Although the resin with high dielectric constant could significantly improve the driving performance of the composite, high dielectric constant resins, such as polyvinylidene fluoride (PVDF), were not often used as the packaging materials for piezoelectric fiber composites because of their weak adhesion property. Besides PFCs, the epoxy resins have been widely employed in various piezoelectric composites. Hwang et al. [15] made a piezoelectric glass fiber reinforced polymer for an impact sensor using the conventional autoclave moulding method, and the ratio of PNN-PZT powder to epoxy resin was 1:10. The ceramic/epoxy-resin composites with 1-3 and 2-2 structures have also been reported and they showed excellent performance [16,17,18]. 

Until now, the effects of viscosity and rigidity of the epoxy resin on PFC’s performance have not been reported. In this paper, three typical epoxy resins were applied in the fabrication of PFC. The effects of epoxy resin properties on the electrical and piezoelectric properties of PFC were studied. The free strain of PFC and the tip displacement of cantilever actuated by PFC were detected. The effect of environmental temperature on the PFC was also discussed.

## 2. Experimental

### 2.1. Preparation of PFC

The PZT ceramics were prepared by viscous plastic processing [19]. PZT-5A presintered powder (Zibo Yuhai Electronic Ceramic Co., Ltd, Zibo, China), solvent, and plasticizer were uniformly mixed to prepare the PZT green body with good flatness [20,21]. The PZT green body was dried in air at 80 °C for four days, which was later sintered at 1200–1300 °C. After sintering, PZT ceramic sheets formed the arrays with a fiber spacing of 80 μm, while the width of fiber was 250 μm. Subsequently, the groove was filled with epoxy resin to form the composite layer. Three different epoxy resins were employed as encapsulating resins, including Araldite 2020 (Huntsman Corp., Everberg, Belgium), DP 460 (3M Corp., Saint Paul, MN, USA), and DP 490 (3M Corp., Cergy-Pontoise, France) resins.

The layer was solidified at 80 °C for 2 h. After curing, it was physically thinned to 200 μm with a lapping machine to obtain the PZT/resin composite. The polyimide films assembled the PZT fibers/resin composites with interdigitated electrodes on the one side. During the encapsulation process, the sample was heated to 80 °C and kept for 0.5 h using a self-made packaging platform. The final obtained piezoelectric fiber composite has a size of 40 × 18 × 0.3 mm^3^ and an effective area size of 28 × 7 × 0.2 mm^3^. In the process of polarization, the piezoelectric fiber composite was dipped in the silicone oil and the polarization electric field was 4 kV/mm. The polarization temperature was 80 °C for 20 min.

### 2.2. Characterization

An impedance analyzer characterized the impedance, phase angle, capacitance, and quality factor of piezoelectric fiber composites (Agilent 4294A, Santa Rosa, CA, USA). A Shore hardness tester characterized the hardness of Araldite 2020, DP 460, and DP 490 epoxy resins (LX-D, HANDPI, Leqing, China), each sample was tested for five times in air at 25 °C, and the average hardness was obtained. 

Strain gauges tested the free strain of piezoelectric fiber composites (Jinan Sigmar Tech Co., Ltd, Jinan, China). A tip displacement test system was built to measure the tip displacement of the cantilever beam, which was driven by the piezoelectric fiber composites. The piezoelectric fiber composite deformed under the actuating voltage, which drove the cantilever beam to move. Laser displacement sensor measured the tip displacement (Micro-epsilon, Passau, Germany). The cantilever beam size was 75 × 28 × 0.45 mm^3^.

## 3. Results and Discussion

### 3.1. Properties of Epoxy Resins

Three different epoxy resins were employed as encapsulating resins, including Araldite 2020 (Huntsman Corp.), DP 460 (3M Corp.), and DP 490 (3M Corp.) resins. Each epoxy resin was made of two components, in which A was resin and B was curing agent. Table 1 shows the characteristics of the three resins. The manufacturers provided the main components, volume ratio, color, and viscosity data of A and B. A LX-D Shore hardness tester measured the hardness.

It can be seen from Table 1 that the hardness of three resins showed little difference, while the main difference existed in the viscosity and color. When the piezoelectric fiber composites were prepared by the cut-and-fill method, the spacing between the PZT fibers was only 50–200 μm. If the viscosity of the resin was too large and the fluidity was too poor, it could easily cause the bubbles when the resin was filled in, which therefore likely increased the risk of breakdown when the PFC was polarized or driven by large voltage.

The curing performance of epoxy resin was also related to the curing conditions [15]. When the curing agent content increased, the crosslinking degree of epoxy resin increased, the heat deflection temperature increased, and the brittleness increased. When the resin cured faster with the increase of the curing temperature, the shear strength became enhanced. The shrinkage rate of the cured epoxy resin was diverse in different curing processes. Moreover, the shrinkage rate increased when the temperature increased, which could even lead to debonding [15]. Those parameters could impact on the performance of piezoelectric fiber composite. The resins that were used in this paper were all commercial resins. According to the formula, they were cured under uniform curing conditions. The curing conditions of the resins were 80 °C for 2 h.

### 3.2. Microstructural Analysis

**Figure 1:**
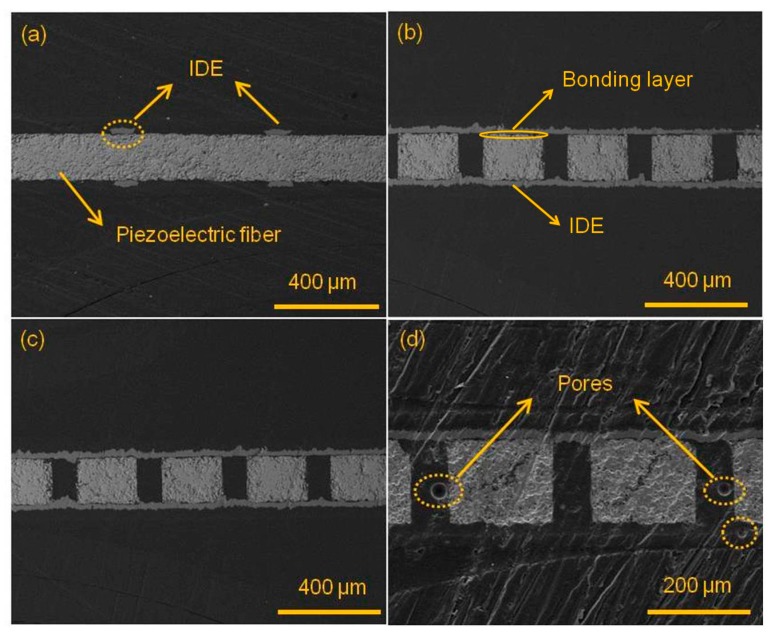
Cross sections of piezoelectric fiber composites (PFC) with different resin (**a**) Araldite 2020, parallel to the fiber length direction; (**b**) Araldite 2020, perpendicular to the fiber length direction, (**c**) DP 460, perpendicular to the fiber direction and (**d**) DP 490, perpendicular to the fiber direction.

The composites that were packaged with epoxy resins Araldite 2020, DP 460 and DP 490 were characterized by scanning electron microscope (SEM), as shown in Figure 1. Figure 1a shows the cross section parallel to the fiber length direction after being packaged with Araldite 2020 epoxy. The PZT fiber with a thickness of 200 μm was sandwiched between the interdigital electrodes and it maintained good integrity and flatness. The upper and lower sides, marked as IDE, were interdigital electrodes with a thickness of 60 μm and width of 90 μm. Figure 1b shows the cross-sectional image perpendicular to the fiber length direction after being packaged with Araldite 2020 epoxy. The brighter square area was PZT ceramic and the dark part between PZT ceramics was Araldite 2020 epoxy. The epoxy resin and PZT ceramic were well bonded. The upper and lower strips of the PZT ceramic were copper electrodes. There was a very thin layer of epoxy between the electrode and PZT, which acted as the bonding layer. Figure 1c,d display the SEM images perpendicular to the fiber length direction when the piezoelectric fiber composites were encapsulated using DP 460 and DP 490, respectively, and the situation is substantially similar to that of Figure 1b. However, it should be noted that the DP 490 epoxy phase in Figure 1d had pores. Among the three types of epoxy resins, the viscosity of the DP 490 epoxy resin was too large, the fluidity was poor, and the wetting angle with the ceramic was large, so that it was difficult to uniformly fill in the gaps between fibers, even in a vacuum environment. The presence of bubbles made the piezoelectric fiber composites tend to local breakdown during large electric field polarization and driving since the breakdown strength of air was very low, i.e., about 2 kV/mm, which affected the strain and driving performance, and even caused the complete failure. Therefore, the performance of PFC with DP 490 was the worst.

### 3.3. Impedance Analysis

**Figure 2:**
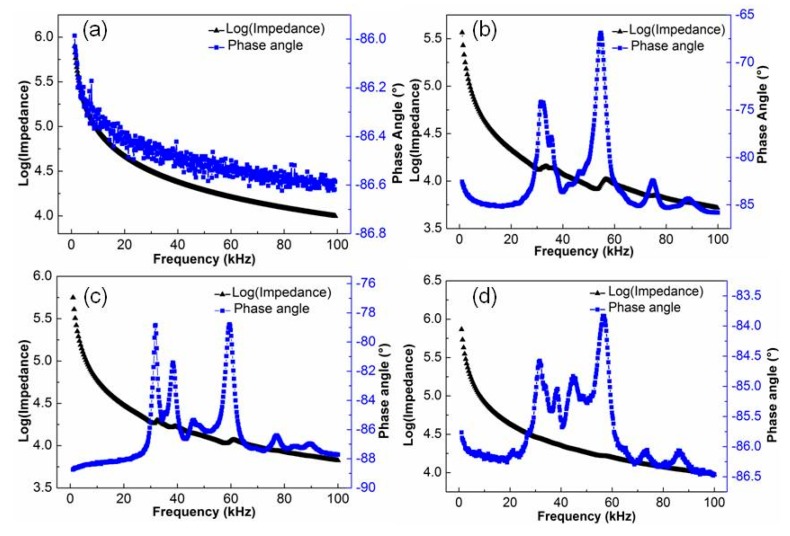
Impedance spectrum of PFC with different epoxy resin (**a**) Araldite 2020 before polarization, (**b**) Araldite 2020 after polarization, (**c**) DP 460 after polarization, and (**d**) DP 490 after polarization

Araldite 2020, DP 460, and DP 490, respectively, packaged the piezoelectric fiber composites. Figure 2 shows the impedance-phase angle spectra. Figure 2a,b reflect impedance-phase angle spectra of the Araldite 2020 resin-packaged piezoelectric fiber composites before polarization and after 5 kV/mm polarization. Before polarization, the impedance of the piezoelectric fiber composite decreases with increasing frequency (Figure 2a), and no resonance peak appears. The phase angle changes little, and it was concentrated at about −87°. The significant peaks of impedance were not obvious. The polarized PFC material exhibits a main resonance peak at the frequency of about 58 kHz (Figure 2b), and the corresponding maximum phase angle difference Δθ was 20°. It was shown that the piezoelectric fiber composite had piezoelectric properties after polarization. When compared with pure PZT ceramics, the Δθ value was significantly reduced, indicating that the degree of polarization was significantly reduced. It was caused by two factors. First of all, the PZT fiber was segmentally polarized along the length direction, and there existed certain inactive regions in the ceramic [13], which led to a decrease in the polarizability of the ceramic. Moreover, the resin layer acted as a capacitor, sharing a large portion of the voltage, due to the encapsulation resin between the electrode and the PZT fiber [13], resulting in a lower effective electric field being applied on the PZT fiber.

The impedance-phase angle spectrum curves of the piezoelectric fiber composites of the DP 460 and DP 490 packages are shown in Figure 2c,d, respectively. The PFCs were encapsulated by using different resins, and the resonance frequencies were generally unchanged, but the phase angle difference Δθ obviously changed. The Δθ values of PFC with DP 460 and DP 490 were 10° and 3°, respectively.

For piezoelectric materials, the resonant frequency has the following relationship with its physical dimension [22]:

(1) $$2f_{r}\cdot l=1/\sqrt{\rho \cdot S^{E}}$$

where *l* was the size factor of the piezoelectric material, *f_r_* was the resonant frequency, *ρ* was the material density, and *s^E^* was material compliance coefficient. *f_r_*·*l* was also called the frequency constant N. For rod-shaped or fibrous piezoelectric materials, when being polarized along the length, its frequency constant was called *N_33_*, and

*N_33_ = f_r_·l* (2)

In Equation (2), *l* was the length of rod-shaped or fibrous piezoelectric material.

Both *ρ* and *s^E^* were the intrinsic parameters of the piezoelectric material, so N_33_ was fixed value. For piezoelectric fiber composites, the size of PZT fiber as the piezoelectric phase was constant and N_33_ was a constant value, so the resonance frequency was constant and independent of the resin.

The viscosity of DP 460 and DP 490 resin may be large in terms of phase angle difference. Under the same packaging pressure, when the viscosity of the resin became larger, and the fluidity became worse, the processability of the encapsulating resin layer became more difficult. Therefore, the encapsulating resin layers of DP 460 and DP 490 were thicker and less polarized than Araldite 2020. It was noted that the piezoelectric fiber composite of the DP 490 package had a Δθ value of only 3° and a very weak polarization. It can be seen from Figure 1d that localized breakdown may occur during the polarization process due to the presence of bubbles in the resin.

### 3.4. Capacitance and Quality Factor Analysis

**Figure 3:**
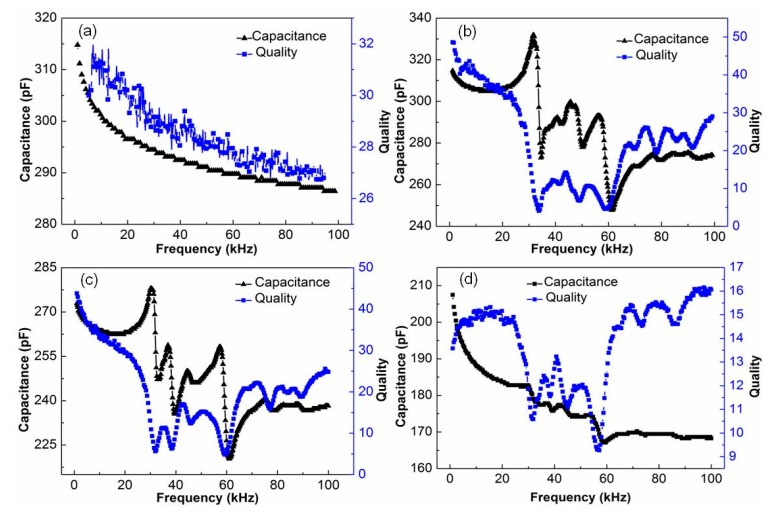
Capacitance-quality spectrum of PFC with three resins. (**a**) Araldite 2020 before polarization, (**b**) Araldite 2020 after polarization, (**c**) DP 460 after polarization and (**d**) DP 490 after polarization.

Figure 3 shows the capacitance-quality factor spectrum of piezoelectric fiber composite with Araldite 2020, DP 460, and DP 490 packages. Figure 3a,b show the capacitance-quality factor spectra of the Araldite 2020 resin-packaged piezoelectric fiber composite before polarization and after 5 kV/mm polarization. Figure 3a shows that both the capacitance and the quality factor of the unpolarized piezoelectric fiber composite decrease with increasing frequency and there was no peak. As can be seen from Figure 3b, the capacitance and quality factor of the polarized piezoelectric fiber composite at 1 kHz were 315.6 pF and 48.2, respectively. The trend with frequency was similar to that of pure piezoelectric ceramics.

Figure 3c,d show the capacitance-quality factor spectrum of the piezoelectric fiber composite after polarization by DP 460 and DP 490, respectively. The capacitance and quality factor at 1 kHz were 272.3, 208.2 pF, and 44.3, 14.8, respectively. The trend with frequency changes was similar to Figure 3b, except that the capacitance and quality factor were reduced. This was consistent with the variation of the phase angle difference and also due to the difference in resin viscosity and fluidity.

### 3.5. Strain and Actuating Analysis

**Figure 4:**
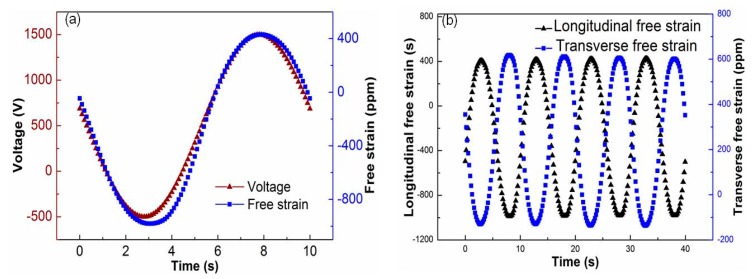
The strain performance of PFC under sine voltage. (**a**) Voltage and strain, (**b**) longitudinal and transverse free strain.

The free-strain test of the piezoelectric fiber composite was carried out at room temperature while using the free strain test system. The selected sample was piezoelectric fiber composites of Araldite 2020 resin package. The test conditions were −500~+1500 V sinusoidal voltage, 0.1 Hz. Figure 4a shows the relationship between the excitation voltage and free strain. As can be seen from the figure, the strain curve and voltage of the piezoelectric fiber composite were both sinusoidal. This was because the domain switching inside the piezoelectric material takes time, so its strain tends to lag behind the applied voltage signal. Figure 4b shows the longitudinal and transverse free strain of the piezoelectric fiber composite. It can be seen from the figure that the longitudinal and transverse strains were consistent with the voltage curve and they were all sinusoidal. The transverse direction was opposite to the longitudinal strain direction and it has a significant driving anisotropy. The longitudinal strain value was approximately 1420 ppm (parts per million) and the transverse strain value was approximately −700 ppm, which was approximately −1/2 of the longitudinal strain value. The longitudinal strain utilizes the d_33_ effect of the PZT piezoelectric fiber and it thus has a large strain value, while the transverse strain utilizes the d_31_ effect of the PZT fiber.

**Figure 5:**
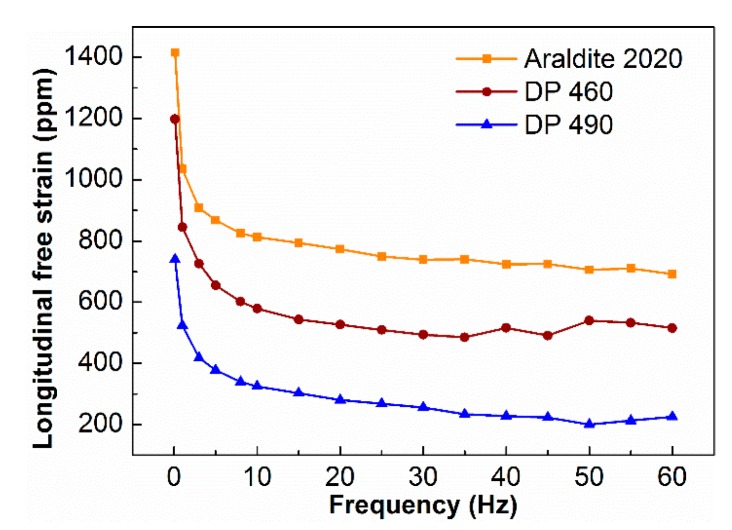
Longitudinal free strain of PFC with different epoxy resin.

The longitudinal free strain tests were carried out on piezoelectric fiber composites that were encapsulated with Araldite 2020, DP 460 and DP 490 epoxy at room temperature. The driving voltage was −500~+1500 V at 0.1~60 Hz. Figure 5 shows the longitudinal free strain of PFC with different epoxy resin. As the frequency increases, the free strain of the PFC sharply drops. When the frequency was higher than 5 Hz, the strain decreases sharply and it tends to be stable. The piezoelectric fiber composites that were packaged with Araldite 2020 epoxy resin had a free strain value of 1420 ppm at 0.1 Hz and a drop of 8.7 ppm at 3 Hz, with a drop of 38.7%. At 60 Hz, the free strain value was 690 ppm; it dropped by 20.7% as compared to the value at 5 Hz. The other two resin-packaged piezoelectric fiber composites had similar trend. At low frequencies, the domains in the piezoelectric material had sufficient time to completely switch and the strain was large. However, as the frequency increased, the domain switching could not keep up with the inversion of the input voltage, the domain incompletely switched, resulting in a smaller strain and eventually stabilized [23,24].

Figure 5 also shows that the free strain of PFCs distinguish with different resin packages. The Araldite 2020 packaged piezoelectric fiber composite had a free strain of 1420 ppm at 0.1 Hz, while the free strain of PFCs packaged with DP 460 and DP 490 were 1200 ppm and 750 ppm under the same conditions. As the volume fraction of the three resins was consistent and the mechanical properties were not much different, the structural parameters of the PZT fibers were also consistent. It was generally related with the degree of polarization for the PZT piezoelectric fibers. The PZT fibers with the Araldite 2020 package had a higher degree of polarization; therefore, it had better performance when actuated by the same voltage. This was accordant with the trend of the impedance-phase angle of Figure 2. 

**Figure 6:**
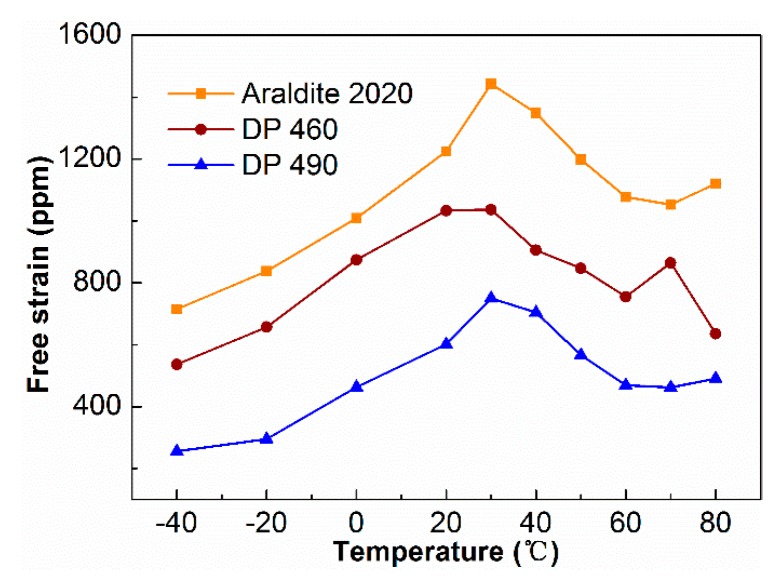
Free strain of PFC at different temperatures.

The piezoelectric fiber composites with different epoxy resin packages were tested for free strain at different temperatures. The excitation voltage was −500~+1500 V, the bias voltage was 500 V, the frequency was at 0.1 Hz, and test temperature range was −40~+80 °C. Figure 6 shows the result. The three resin-packaged piezoelectric fiber composites had similar trends in the temperature range of −40 to +80 °C. In piezoelectric fiber composites with Araldite 2020, the free strain increases from low temperature to room temperature, which was 730 ppm at −40 °C, and reaches the maximum of 1440 ppm at 30 °C. Above 30 °C, the free strain value decreased and the lowest value was 1050 ppm, and the descend range was 27.1%. The situation at high temperature was consistent with the simulation data of Inman [25]. Therefore, the effect of temperature was significant on the strain of the piezoelectric fiber composite.

At lower or higher temperatures, the strain properties of the piezoelectric fiber composites were both significantly reduced. The main reason was the difference in the thermal expansion coefficients of various materials in the composite [25]. The thermal expansion coefficient of PZT ceramics was about 2.5~5.5×10^−6^/°C [26], while the thermal expansion coefficient of epoxy resin polymer was about 50~60×10^−6^/°C [25]. The difference was an order of magnitude. When the piezoelectric fiber composite was put in a low temperature or high temperature environment, the difference in the thermal expansion coefficient between the ceramic phase and the resin phase became larger, and the deformation inconsistently occurs, resulting in internal stress inside the piezoelectric fiber composite, even prompting the peeling to occur between the fiber and the epoxy resin, therefore the strain of the composite decreased. Moreover, the activity of piezoelectric materials was lower at low temperatures, which was also an important reason for the poor performance.

**Figure 7:**
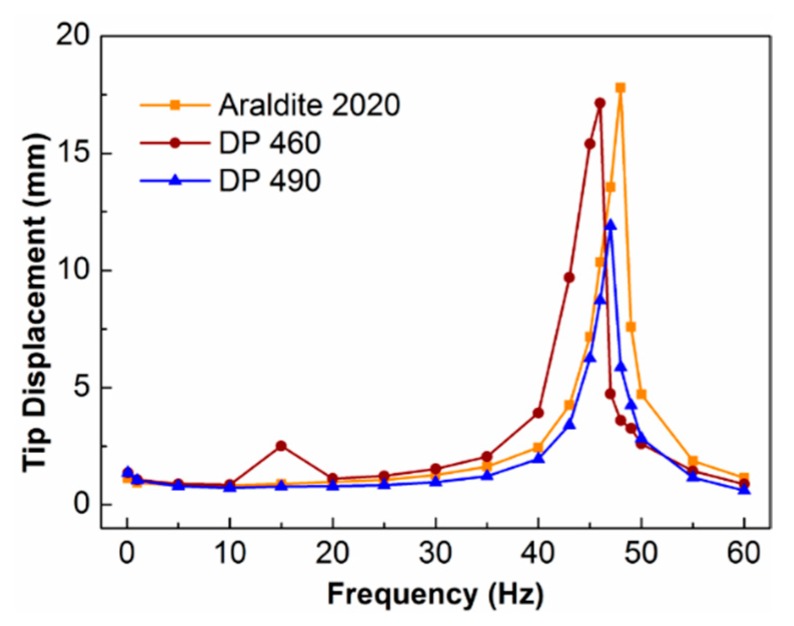
Tip displacement of cantilever beam actuation by PFC with different epoxy resin.

The piezoelectric fiber composites of the Araldite 2020, DP 460, and DP 490 packages were attached to the steel cantilever beam, and the tip displacement values of the cantilever beams were tested while using the test system. The conditions of driving voltage were −500~+100 V, offset 500 V at 0.1~60 Hz. Figure 7 shows the curve of the tip displacement with frequency. The relationship between the tip displacement of the cantilever beam that was driven by the three resin-packaged piezoelectric fiber composites was basically the same. When the frequency was less than 40 Hz, the tip displacement did not change significantly with frequency; when the frequency was 40~50 Hz, the tip displacement sharply increased first and then sharply decreased; when the frequency was above 50 Hz, the tip displacement decreased to the initial level. Between 45 to 47 Hz, there was a maximum value of the tip displacement, which was called the resonance frequency. When Araldite 2020 was used as the encapsulating resin, the maximum value was 17.8 mm, and when DP 460 and DP 490 were used as encapsulating resins, the maximum values were 17.1 and 11.9 mm, respectively. The performance of the piezoelectric fiber composite was poor, as the Δθ value of DP 490 packaged composites was only 3° (Figure 2d), due to the viscosity and fluidity of the resin. The tip displacement of cantilever driving by the PFC with DP 490 was lower than the others. It shows that, when the piezoelectric fiber composite material was encapsulated with Araldite 2020 resin, it had large driving performance.

## 4. Conclusions

In this paper, piezoelectric fiber composites were filled and encapsulated with three epoxy resins, Araldite 2020, DP 460, and DP 490. The viscosity of the three resins was significantly different, but the mechanical properties were not much different. The epoxy resin could be well bonded with the piezoceramic fiber. However, the DP 490 with the highest viscosity led to bubbles in the PFC, which affected the polarization and driving performance. Being packaged with Araldite 2020, the longitudinal free strain and the lateral free strain reached 1420 ppm and −700 ppm, respectively, with significant anisotropy. When the temperature of environment was −40 to +80 °C, the free strain value increased at first and then decreased with increasing the temperature, reaching the maximum of 1440 ppm at 30 °C. The other two epoxy resin-packaged piezoelectric fiber composites followed the same rule. With Araldite 2020, the free strain and the tip displacement values of PFC were 1420 ppm and 17.8 mm, respectively, and the strain and drive performance were optimal. Therefore, the viscosity of epoxy resin demonstrated very important roles in the performance of piezoelectric fiber composites, and epoxy resin with similar properties to Araldite 2020 was better choice.

## Figures and Tables

**Table 1:** Physical and chemistry properties of epoxy resin.

Epoxy Resin	Component	Mass Ratio (A:B)	Colour	Viscosity (Pa·s)	Shore Hardness (HD)
Araldite 2020	A: Bisphenol A-(epoxy chloropropane) B: 1,4-bis(2,3-epoxypropoxy)butane	10:3	transparent	0.15	75.9 ± 0.5
DP 460	A: Tris(2,4,6-tris(dimethylamino)methyl)phenol B: 4,7,10-trioxacyclohexane-1,13-diamine	2:0.96	White	25	75.0 ± 0.5
DP 490	A: 4,4’-diphenolyl propane epichlorohydrin B: 1,4 -bis((2,3-epoxypropoxy)methyl)cyclohexyl	2:1	Black	250	79.2 ± 0.3
